# Mental well-being among Syrian refugee workers in Lebanon: a multidimensional approach

**DOI:** 10.7189/jogh.16.04086

**Published:** 2026-03-20

**Authors:** Rima R Habib, Ghida Al Nakib, Lina M Fakih, Zeinab Awad, Lea Saad, Fida Awada, Mira F Kanaan, Iman Nuwayhid

**Affiliations:** Department of Environmental Health, American University of Beirut, Beirut, Lebanon

## Abstract

**Background:**

Displaced populations endure structural, psychological, and social vulnerabilities that impact their mental well-being. Lebanon’s economic collapse, political instability, and inadequate infrastructure have exacerbated the hardships of its 1.5 million Syrian refugees. In this study, we applied the Six Dimensions of Wellness framework to explore the factors influencing the mental well-being of a sample of Syrian refugee workers in Lebanon.

**Methods:**

We conducted a cross-sectional analysis on 89 Syrian refugee agricultural workers (42 men and 47 women) recruited from 32 greenhouse farms in Lebanon’s Beqaa Valley. Participants completed structured questionnaires covering the Six Dimensions of Wellness. Mental well-being was assessed using the World Health Organization five well-being index, and scores below 13 indicated poor well-being. We used descriptive statistics and logistic regression to examine associations between wellness indicators and mental well-being.

**Results:**

We found that female workers, compared to male workers, reported a notably higher prevalence of poor mental well-being (40.4% *vs.* 23.89%). Participants who had experienced a work-related injury had nine times higher odds of poor mental well-being than those who had not (adjusted odds ratio (aOR) = 9.30; 95% confidence interval (CI) = 1.64–52.79). Social support was protective of mental well-being (aOR = 0.52; 95% CI = 0.28–0.97), while having difficulty accessing health information (aOR = 6.09; 95% CI = 1.61–23.06) and unmanageable work demands (aOR = 10.09; 95% CI = 2.47–41.31) were significantly linked to poor mental well-being. Although protective against poor well-being, results for age and education were not statistically significant.

**Conclusions:**

This study highlights the complex and multidimensional nature of mental well-being among Syrian refugee agricultural workers. Work injuries, lack of access to health information, and high work demands increase the odds of poor mental well-being, while social support offers protection. Our findings inform interventions to improve mental well-being for displaced populations.

Forced migration, often driven by conflict and instability, has profound implications for global health, sustainable development, and social cohesion [1,2]. Over one billion people worldwide are currently on the move, including 84 million forcibly displaced individuals [3]. These populations, particularly refugees, face complex challenges stemming from displacement itself and systemic barriers in host societies [3,4]. As a result, they experience heightened risks of anxiety, depression, psychological distress, and chronic and infectious diseases compared to host populations [5,6]. Lebanon exemplifies an acute intersection of migration, well-being, and socioeconomic fragility. Hosting approximately 1.5 million Syrian refugees, it maintains one of the world’s highest refugee-to-population ratios [7]. Prolonged economic collapse, political paralysis, and the COVID-19 pandemic have severely strained the country’s capacity to support both citizens and displaced communities [8]. The Lebanese pound’s depreciation of over 90% has driven widespread poverty, while limited access to electricity, healthcare, clean water, and the devastation of the 2020 Beirut port explosion have further weakened living conditions [9]. The protracted displacement of Syrian refugees in Lebanon’s overcrowded informal settlements perpetuates conditions of stress and psychological disorders [9,10].

Among Syrian refugees in Lebanon, mental health issues, particularly anxiety, depression, and trauma-related symptoms, are closely linked to poverty, legal insecurity, and social exclusion [11,12]. With around 90% of refugee households living in extreme poverty [13], inflation and currency devaluation have rendered basic necessities unaffordable, forcing families into harmful coping strategies such as child labour, early marriage, and reduced healthcare spending [14]. When available, employment is often informal and exploitative, contributing to persistent vulnerability [15]. Although the United Nations High Commissioner for Refugees facilitates access to healthcare, funding shortfalls have led to reduced coverage [16,17]. On the other hand, community solidarity and informal support networks offer some resilience [11,18], yet these are insufficient substitutes for comprehensive, inclusive policies that prioritise refugee mental well-being [11]. Amid Lebanon’s compounded crises and its high refugee-to-population ratio, examining the mental well-being of Syrian refugees is essential to uncover how physical, psychological, and social factors interact to shape resilience and overall well-being.

## Conceptual framework

**Figure 1:**
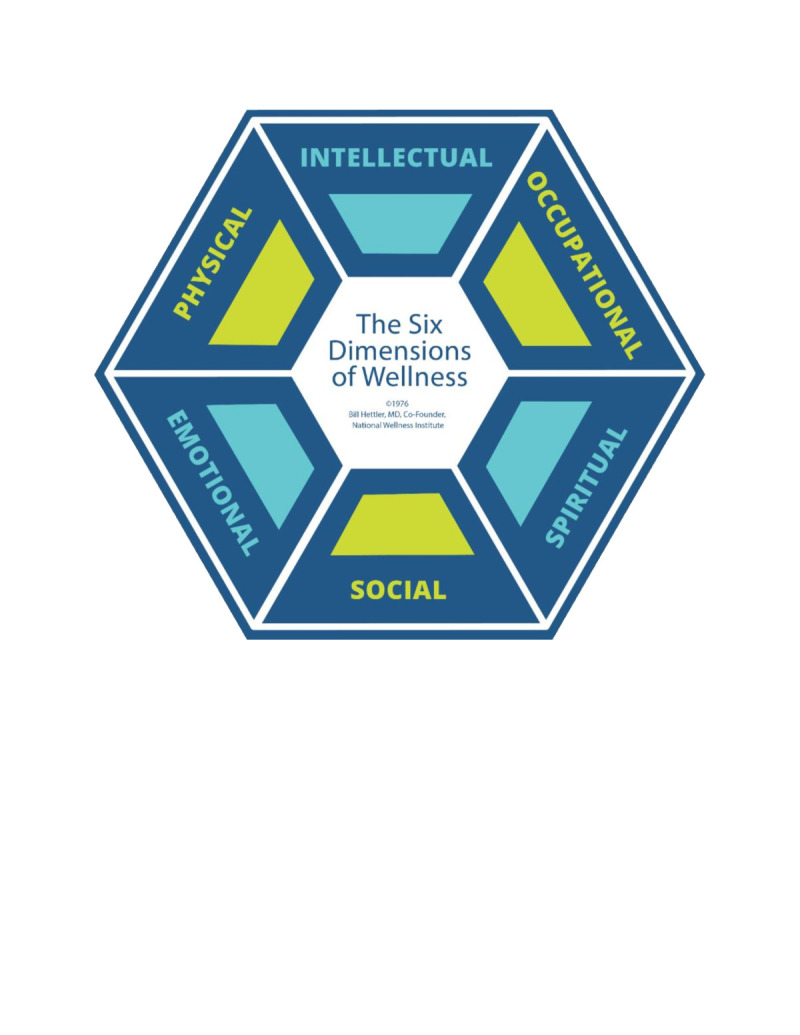
The Six Dimensions of Wellness.

Our analysis of mental well-being among Syrian refugees in Lebanon was guided by Bill Hettler’s Six Dimensions of Wellness framework, which encompasses the physical, mental, emotional, social, spiritual, and occupational domains [19] (**Figure 1**). This integrative model offers a comprehensive and flexible lens for assessing well-being, capturing the multidimensional social, economic, physical, and psychological factors that shape mental well-being among displaced populations [11]. Unlike models that focus predominantly on either psychological or structural determinants, Hettler’s framework accommodates the complex realities of displacement, such as prolonged uncertainty, disrupted social networks, and limited access to essential resources, by consolidating multiple domains of well-being into a single, adaptable model [20,21]. This framework has also been widely applied in low- and middle-income countries (LMICs), settings that share important structural similarities with refugee-hosting contexts [22-27].

In addition, this framework recognises the interconnectedness of individual and social dimensions of wellness and is both adaptable and well-established in LMIC contexts, enabling a nuanced understanding of how forced displacement and ongoing instability shape mental well-being. To ensure cultural relevance and validity, we reviewed and contextually adapted the indicators within each domain while preserving the framework’s theoretical integrity and alignment with the lived realities of Syrian refugees in Lebanon.

## Research objectives

For Syrian refugees in Lebanon, mental well-being is deeply intertwined with the multiple hardships they experience throughout the displacement continuum, from forced migration to prolonged settlement in precarious conditions [4]. However, existing research has predominantly focused on mental health outcomes with limited attention to broader, multidimensional contributors to mental well-being [4,28]. In this study, we explored how various dimensions of wellness, including physical, social, intellectual, spiritual, emotional, and occupational, intersect to shape the mental well-being of Syrian refugee agricultural workers in Lebanon’s Beqaa Valley. Addressing this gap is essential to informing culturally grounded, equity-oriented strategies that strengthen resilience and improve quality of life in contexts of protracted displacement.

## METHODS

### Study design

This analysis is part of the larger Global Environmental and Occupational Health Research and Training Hub for the Middle East and North Africa (GEOHealth-MENA) project, funded by the National Institutes of Health, which aims to examine a number of occupational exposures and their effects on health outcomes among Syrian refugee workers in the Beqaa Valley of Lebanon. For this paper, a cross-sectional analysis was conducted to examine associations between indicators of well-being, as defined by the Six Dimensions of Wellness framework [29], and poor mental well-being among Syrian refugee workers. Eligible participants were 89 Syrian men and women agricultural workers aged between 18–55 years. Workers were excluded if they reported neurological or psychiatric conditions, long-term psychotropic medication use, head injury, or other conditions that could influence mental well-being or neurobehavioral function.

### Recruitment and data collection

Data collection took place within a 10 km radius of the American University of Beirut’s extension centre, known as ‘advancing research enabling communities’ (ARECs), located in the Beqaa Valley, in an area where greenhouse farms employing Syrian agricultural workers are widely spread [30]. We collected data between 29 July and 23 September 2024. We recruited male and female Syrian refugee agricultural workers from 32 farms with at least 10 greenhouses, planting crops such as tomatoes, cucumbers, and peppers [30]. After obtaining the farm owners’ consent, all Syrian workers present at the farm during the research team’s field visits were approached, consented to, and screened for eligibility. When the number of eligible workers exceeded two per gender, we randomly selected two men and two women using the SurveyCTO software, version 2.81 (Dobility Inc., Cambridge, Massachusetts, USA), a data collection platform installed on the data collector’s tablets to administer eligibility forms and study questionnaires [31].

Institutional Review Board at the American University of Beirut (BIO-2022-0376) approved this study. We obtained oral consent from all participants in the study.

### Study instruments and measures

Participants were interviewed face-to-face by trained field staff using structured questionnaires in colloquial Arabic. Questionnaires were pre-tested for contextual relevance among Syrian refugee workers in a similar neighbourhood in the Beqaa Valley and included sections on chronic illnesses, work-related injuries, social support, educational level, access to medical and health information, religious engagement, life satisfaction, work burden, and discrimination; based on our previous research experience in the same neighbourhood [32].

Our analysis was based on a set of indicators reflecting the components of the Six Dimensions of Wellness framework (**Figure 1**). These indicators were relevant to the study’s context and were pilot-tested on a sample of Syrian refugees living and working in a nearby neighbourhood in Beqaa.

We used chronic diseases and work-related injuries as measures of the physical wellness domain. The chronic diseases section included 18 chronic conditions (yes/no). We categorised chronic diseases as having none vs having at least one chronic disease. We assessed work-related injuries as having ever experienced a work-related injury (yes/no). We used the Medical Outcomes Study Social Support Survey [33], including 19 items, as a proxy for the social wellness domain. Each item was scored on a scale from one ‘None of the time’ to five ‘All the time.’ The overall index was obtained by averaging all 19 items, generating a total score ranging from one (no social support) to five (continuous social support) [34]. We evaluated the intellectual wellness domain by the participants’ years of formal education and their ability to access and understand health-related information independently. To assess the spiritual wellness domain, participants were asked about their attendance at religious meetings (none or at least once a year). The overall life satisfaction question (satisfied/dissatisfied) was used as a proxy for the emotional well-being domain. We assessed the occupational wellness domain through the Chronic Work Discrimination and Harassment (CWDH-a) scale [35]and a question on work burden (having enough time to do excessive work). The CWDH-a scale includes five items ranked from one ‘never’ to five ‘once a week or more.’ The total score is obtained by summing up all items, and ranges from five (no work discrimination and harassment) to 25 (almost daily work discrimination and harassment) [36]. The work burden is assessed using a single item from the Karasek job scale, ‘Having enough time to manage an excessive amount of work’ (agree/disagree) [37].

We assessed mental well-being as the outcome of interest using a validated scale in Lebanon among Syrian refugees – World Health Organization (WHO) five well-being index – which consists of five items rated from zero ‘at no time’ to five ‘all the time’ [38,39]. The total score is obtained by summing up all items, and it ranges from 0–25. We used a cut-off of 13 to categorise participants into poor mental well-being (<13) and good mental well-being (≥13) [40].

### Data analysis

We used descriptive statistics to summarise the study variables. Frequencies and percentages were reported for categorical variables, while means and standard deviations (SD) were calculated for continuous variables. Normality was assessed using the Shapiro-Wilk test. For the descriptive analysis, we examined gender differences using the independent *t* test for normally distributed variables, the Mann-Whitney rank-sum test for skewed variables, and the χ^2^ test for categorical variables.

We conducted bivariable analyses to explore associations between each covariate and mental well-being. We performed multiple logistic regression to examine associations between mental well-being and the selected covariates. We assessed multicollinearity using the uncentered variance inflation factor, and model fit was evaluated using the ‘estat gof’ command. We conducted analyses using Stata, version 17.0 (StataCorp LLC, College Station, Texas, USA) [41]. We considered a *P*-value <0.05 to be statistically significant.

## RESULTS

We screened a total of 163 participants (63 male and 100 female workers) for eligibility. Of these, 111 were deemed eligible, and 104 (49 male and 55 female) workers provided consent to participate in the study. One male worker refused to participate, and 13 did not complete the study (five male and eight female workers). Of the 90 workers who completed the study, one was excluded from the analysis due to a missing value on the variable ‘overall life satisfaction’, which is part of the emotional domain of the framework. The analysis included 89 workers (42 men and 47 women).

The participants had a mean age of 33.5 years (SD = 10.5); male and female workers were nearly equally represented (47.2% and 52.8% respectively). Poor mental well-being was more frequent among female than male workers (40.4% vs 23.8%). Over half (55.1%) reported no chronic disease, though a greater proportion of females had one or more chronic conditions (53.2% vs 35.7%). Work-related injuries were more commonly reported by males (23.8% vs 8.5%). The mean social support index was 3.2 (SD = 1.0), and the average years of education were 4.7 years (SD = 3.6). Access to health information was lower among females (46.8%) than among males (61.9%). Most participants (83.2%) did not attend religious meetings, and more than two-thirds (71.9%) reported being satisfied with life. The mean score for chronic work discrimination and harassment was 10.4 (SD = 5.3), and just over half (56.2%) reported having enough time to manage work demands (**Table 1**).

**Table 1:** Bivariate analysis comparing male and female Syrian refugee agricultural workers (aged 18–55 y) on mental well-being and domains of the Six Dimensions of Wellness, Beqaa, Lebanon, 2024 (n = 89)*

Items	Total	Male workers	Female workers	*P*-value
Total	89 (100.0)	42 (47.2)	47 (52.8)	
Mental well-being†				0.095
*Good*	60 (67.4)	32 (76.2)	28 (59.6)	
*Poor*	29 (32.6)	10 (23.8)	19 (40.4)	
Mental well-being score, x̄ (SD)	15.6 (6.1)	16.9 (5.0)	14.5 (6.7)	0.058
Age in years, x̄ (SD)	33.5 (10.5)	32.9 (11.1)	34.1 (10.0)	0.565
Physical domain: having a chronic disease				0.098
*None*	49 (55.1)	27 (64.3)	22 (46.8)	
*At least one*	40 (44.9)	15 (35.7)	25 (53.2)	
Physical domain: ever had a work-related injury				0.048
*No*	75 (84.3)	32 (76.2)	43 (91.5)	
*Yes*	14 (15.7)	10 (23.8)	4 (8.5)	
Social domain: social support index, x̄ (SD)	3.2 (1.0)	3.0 (1.1)	3.3 (0.9)	0.130
Intellectual domain: education in years, x̄ (SD)	4.7 (3.6)	5.1 (3.3)	4.3 (3.8)	0.303
Intellectual domain: access to health or medical information				0.154
*I can understand and access information*	48 (53.9)	26 (61.9)	22 (46.8)	
*I need help with access to information*	41 (46.1)	16 (38.1)	25 (53.2)	
Spiritual domain: attending religious meetings				0.098
*Never*	74 (83.2)	32 (76.2)	42 (89.4)	
*At least once a year*	15 (16.8)	10 (23.8)	5 (10.6)	
Emotional domain: overall life satisfaction				0.073
*Satisfied*	64 (71.9)	34 (81.0)	30 (63.8)	
*Dissatisfied*	25 (28.1)	8 (19.1)	17 (36.2)	
Occupational domain: chronic work discrimination and harassment score, x̄ (SD)	10.4 (5.3)	10.4 (4.8)	10.4 (5.7)	0.799
Occupational domain: having enough time to do an excessive amount of work				0.548
*Agree or strongly agree*	50 (56.2)	25 (59.5)	25 (53.2)	
*Disagree or strongly disagree*	39 (43.8)	17 (40.5)	22 (46.8)	

SD – standard deviation, x̄ – mean

*Presented as n (%) unless specified otherwise.

†Poor mental well-being defined as WHO-5 score <13.

We used bivariate analysis followed by multiple logistic regression to examine associations with poor mental well-being (**Table 2**). Although the statistical significance differed for some variables after adjustment, the direction of the odds ratios (ORs) remained consistent between the unadjusted and adjusted models, except for ‘attending religious meetings’ (OR = 0.46, 95% confidence interval (CI) = 0.12–1.78; adjusted OR (aOR) = 1.15, 95% CI = 0.17–7.64).

**Table 2:** Unadjusted and adjusted odds ratios for age, gender, and domains of the Six Dimensions of Wellness with poor mental well-being among Syrian refugee agricultural workers (18–55 y), Beqaa, Lebanon, 2024 (n = 89)

Characteristics	Poor mental well-being, n = 29 (32.6%)			
	OR (95% CI)*	***P*-value**	**aOR (95% CI)†**	***P*-value**
Age in years	0.99 (0.95–1.03)	0.558	0.97 (0.92–1.03)	0.381
Gender				
*Male*	ref		ref	
*Female*	2.17 (0.87–5.44)	0.098	3.32 (0.91–12.11)	0.069
Physical domain: having a chronic disease				
*None*	ref		ref	
*At least one*	2.28 (0.92–5.63)	0.074	2.15 (0.64–7.16)	0.214
Physical domain: ever had a work-related injury				
*No*	ref		ref	
*Yes*	2.41 (0.76–7.68)	0.137	9.30 (1.64–52.79)	0.012
Social domain: social support index	0.73 (0.47–1.14)	(0.164)	0.52 (0.28–0.97)	0.039
Intellectual domain: education in years	0.96 (0.85–1.09)	0.548	0.97 (0.81–1.15)	0.712
Intellectual domain: access to health or medical information				
*I can understand and access information*	ref.		ref	
*I need help with access to information*	3.28 (1.30–8.30)	(0.012)	6.09 (1.61–23.06)	0.008
Spiritual domain: attending religious meetings				
*Never*	ref		ref	
*At least once a year*	0.46 (0.12–1.78)	(0.262)	1.15 (0.17–7.64)	(0.884)
Emotional domain: overall life satisfaction				
*Satisfied*	ref		ref	
*Dissatisfied*	2.55 (0.98–6.67)	(0.056)	3.06 (0.86–10.85)	(0.084)
Occupational domain: chronic work discrimination and harassment score	1.06 (0.97–1.15)	(0.200)	1.04 (0.93–1.17)	(0.480)
Occupational domain: having enough time to do an excessive amount of work				
*Agree or strongly agree*	ref		ref	
*Disagree or strongly disagree*	3.8 (1.49–9.68)	0.005	10.09 (2.47–41.31)	0.001

AOR – adjusted odds ratio, CI – confidence interval, OR – odds ratio, ref – reference

*Unadjusted logistic regression.

†Logistic regression adjusting for age, gender, and all variables shown in the table.

In the adjusted model, participants with a work-related injury (aOR = 9.30; 95% CI = 1.64–52.79) had higher odds of poor mental well-being. Greater social support was protective (aOR = 0.52; 95% CI = 0.28–0.97), while difficulty accessing health information (aOR = 6.09; 95% CI = 1.61–23.06) and inability to manage excessive work demands (aOR = 10.09; 95% CI = 2.47–41.31) were associated with poorer mental well-being. Those who showed dissatisfaction with life were three times more likely to report poor mental health (aOR = 3.06; 95% CI = 0.86–10.85), however this finding did not reach statistical significance. No significant associations were found in the adjusted model for participants with chronic disease, or for variables such as education, religious participation, and work discrimination.

## DISCUSSION

In this study, we examined the association between multiple dimensions of wellness, physical, social, intellectual, spiritual, emotional, and occupational, and mental well-being among Syrian refugee agricultural workers in Lebanon’s Beqaa Valley, using the Six Dimensions of Wellness framework. Findings contribute to the growing research on Syrian refugees’ mental well-being. Overall, 35.5% of participants exhibited poor mental well-being, a prevalence notably higher than in comparable populations; for example, Sharma et al. reported 14.1% among Nepalese migrant workers in the Gulf region [42]. By quantifying poor mental well-being and situating it within post-migration stressors, the study highlights overlooked aspects of refugee mental health and informs policy and context-sensitive interventions. Additionally, the results support Hettler’s framework as a useful tool for understanding interactions among different wellness dimensions.

The gender differences observed in our study highlight the importance of intersectional dynamics in shaping mental well-being among Syrian refugee workers. Descriptive results showed that female workers had a notably higher prevalence of poor mental well-being (43.9%) compared with male workers (26.0%). This aligns with a previous study indicating that stressors experienced by refugee workers differ across gender [43]. Particularly, physical factors such as work-related injuries were significantly higher among male workers. Existing evidence suggests that this difference may reflect the gendered organisation of agricultural labour [44]. In agricultural settings, tasks are often allocated by gender, with male workers more frequently engaged in activities such as spraying, operating machinery, and transporting crops, while female workers are more commonly involved in harvesting and other crop-handling tasks such as sowing and weeding [44,45]. These distinct roles expose workers to different occupational risks, which may contribute to observed variations in injury patterns between male and female migrant workers.

The majority of female workers in our sample reported having at least one chronic disease (a physical domain factor) and difficulty accessing health information (intellectual domain). Although other predictors did not reach statistical significance, notable gender differences were still evident [14]. Female participants generally had fewer years of education, were less likely to attend religious meetings, reported lower life satisfaction, and were more likely to report not having enough time to manage excessive workloads. These patterns are further explained by socially constructed gender norms within Syrian refugee communities, where women often assume additional responsibilities such as household chores and childcare [46], compounding their overall burden. These differences may explain the higher prevalence of poor mental well-being observed among female participants in the study population.

Poor mental well-being in our study population was predicted by several factors across multiple domains: physical (*e.g.* history of work-related injuries), social (*e.g*. low perceived social support), intellectual (*e.g.* limited access to medical and health information), and occupational (*e.g.* insufficient time to manage excessive workloads).

The results of our study align with previous literature on risk factors for poor mental well-being among refugee populations. Existing health issues, workplace stressors, inadequate working conditions, discrimination, reliance on precarious employment and limited social support were all factors previously identified to predict poor mental health in these populations [47]. The mental well-being of Syrian refugees, particularly, has been linked to their living conditions, such as financial hardship, limited social support, discrimination, and legal status [48].

Participants with low perceived social support were more likely to report poor mental well-being, reflecting the well-established role of social support as a protective factor for the mental well-being of Syrian refugees in Lebanon [47]. Although not statistically significant, participants with chronic conditions in our study reported poorer mental well-being, consistent with evidence linking chronic diseases to increased likelihood of poor mental health [49]. Lower life satisfaction was associated with poor mental well-being, aligning with previous studies showing an association between life dissatisfaction and poor mental health [50]. Our analysis further identified occupational factors, particularly ‘not having enough time to do excessive work’, as a significant predictor of poor mental well-being. This supports evidence of a systematic review highlighting that workplace psychosocial stressors and poor working conditions are key contributors to mental health outcomes in refugee workers [51]. The strong association between the inability to manage excessive work demands and poor mental well-being in the study population reflects the realities of refugee workers’ conditions in Lebanon. Refugee agricultural workers operate in the informal sector [52], with limited labour protection and excessive workloads, often working two shifts per day depending on seasonal demands [32,52]. This situation leaves employers unbound from legal obligations to provide these workers with access to healthcare or compensation for work-related accidents [52]. Refugees’ legal status further compounds these issues, imposing restrictions on their mobility [45], limiting their access to formal employment, and increasing the risk of being exploited by employers through minimal wages (as low as USD 10–15 per 8–10-hour workday), increased working hours, and lacking basic legal protections [14,53]. Additionally, intellectual domain factors, particularly requiring assistance in accessing health information, align with prior research showing that limited health literacy can compromise decision-making and contribute to poor mental health [54–56].

The findings of this study indicate that poor mental well-being among Syrian refugee workers is multidimensional and shaped by structural and environmental constraints. In Lebanon, refugee-related policies influence health and well-being, particularly the non-encampment approach, which leaves refugees without formal camps and forces many to reside in overcrowded, underserved informal settlements in the Beqaa [57]. These living conditions – marked by shared, limited resources and inadequate access to public health services – further compound vulnerability. Consequently, interpreting research on Syrian refugees in Lebanon requires moving beyond individual-level vulnerabilities and acknowledging the need for broader policy reforms and a comprehensive public health response that addresses all six dimensions of wellness.

Collectively, these factors underscore that improving mental well-being among Syrian refugee workers requires interventions that address the specific nature of the identified predictors. Strengthening social support systems, enhancing health literacy, and improving working conditions are key issues. Practical measures may include culturally and linguistically appropriate health education materials – such as guidance on coping strategies, workplace safety practices, and harm-reduction approaches [58]. Establishing peer-support groups could also reinforce workers’ coping capacities [58], particularly given that many Syrian refugee workers reside in informal tented settlements or densely connected community settings. Ultimately, lasting progress will depend on addressing the structural roots of these challenges through policy-level reforms that reshape the conditions under which refugee workers are employed. Specifically, policies should prioritise gender-responsive workplace standards, legal and policy reforms to address inequities, and community-level initiatives that support both emotional and occupational wellness. Integrating these intersectional considerations into interventions would improve their relevance and effectiveness for refugee workers facing multiple vulnerabilities.

### Limitations

This study has several limitations. First, restricting farms to those located within a 10 km radius of the AREC centre in the Beqaa Valley may have limited the generalisability of our results beyond the study area. Second, the study is cross-sectional, so causality cannot be inferred. In addition, the small sample size may limit the statistical power and the precision of some estimates. We also encountered 14 non-responses; although replacements were randomly selected in SurveyCTO to reduce volunteer bias, the potential for such bias remains a limitation of this study. Moreover, 13 participants did not complete the study, which could represent non-response bias.

The indicators of wellness and the dependent variable, mental well-being, were self-reported, which could introduce measurement error and potential misclassification bias. The participants’ social and legal vulnerabilities may have influenced their decision to participate and introduced selection bias. Moreover, face-to-face interviews may have led to socially desirable responses. To minimise interviewer bias, questionnaires were translated into the local Arabic dialect to reduce interpretation differences and avoid leading questions. Data collectors received extensive training to maintain a neutral and non-judgmental tone, and participants were reminded that they could choose not to answer questions that made them uncomfortable. Additionally, validated scales and standardised questionnaires were used, and confidentiality was assured to further minimise potential misclassification bias.

Although the WHO-five index has been previously validated in Arabic and used among Syrian refugee populations, cultural differences in how concepts such as mood, energy, and well-being are understood may influence participants’ interpretation and responses to its items. These cultural nuances may introduce variability in how wellness constructs are measured in this study.

## CONCLUSIONS

In this study, we examined the association between poor mental well-being and the Six Dimensions of Wellness outlined in Hettler’s framework among Syrian refugee agricultural workers in Lebanon. Poor mental well-being was associated with three of the six domains – physical health (work-related injuries), intellectual factors (access to health information), and occupational factors (not having enough time to do excessive work). These findings emphasise that structural and psychosocial determinants are required to improve mental well-being among refugee agricultural workers. Work-related injuries, unsafe working conditions, and barriers to information contribute to distress, whereas strong social networks and community ties offer protection.
